# Epidrug Modulated Expression of MiR­-152 and MiR-148a Reverse Cisplatin Resistance in Ovarian Cancer Cells: An Experimental In-vitro Study

**DOI:** 10.22037/ijpr.2020.15450.13217

**Published:** 2020

**Authors:** Sahel Khajehnoori, Fatemeh Zarei, Mahta Mazaheri, Ali Dehghani-Firoozabadi

**Affiliations:** a *Department of Medical Genetics, School of Medicine, Shahid Sadoughi University of Medical Sciences,Yazd, Iran. *; b *Mother and Newborn Health Research Center, Shahid Sadoughi University of Medical Sciences, Yazd, Iran. *; c *Cardiovascular Research Center, Shahid Sadoughi University of Medical Sciences,* *Yazd, Iran.*; 1 * S. K. and F. Z. contributed equally to this work.*

**Keywords:** Ovarian neoplasms, MicroRNAs, DNA (cytosine-5)-methyltransferase 1, Trichostatin A, Azacitidine

## Abstract

Cisplatin is a common agent which is used to treat Epithelial Ovarian Cancer (EOC), but cisplatin resistance is a major obstacle in successful treatment of ovarian cancer. Aberration in epigenetic changes play an important role in disregulation of gene expression. MiR-152 and miR-148a are frequently down-regulated in EOC due to promoter hyper-methylation. DNA methyltransferase1 (DNMT1), the main enzyme in maintenance of the pattern of DNA methylation, is one of the targets of miR-152 and miR-148a. Aberrantly up-regulation of DNMT1 is responsible for silencing of tumor suppressor genes in carcinogenesis. We hypothesized that re-expression of miR-152 and miR-148a and consequently down-regulation of DNMT1 may resensitize cancerous cells to chemotherapeutics agents. The aim of the present study is to investigate the effect of 5-azacytidine (5-Aza) and Trichostatin A on miR-152 and miR-148a expression in A2780CP ovarian cancer cell line. Optimal doses of 5-Azacitidine and TSA were measured by 3-(4,5-dimethylthazol-2-yl)-2,5-diphenyltetrazolium bromide (MTT) assay. A2780CP cell line was treated by each drugs, alone or in combination and the expression of miR-148a, miR-152 and DNMT1 was evaluated by *Real*-*Time Quantitative* *Reverse Transcription*-*Polymerase Chain Reaction (RT-qPCR).* The results revealed that TSA and 5-Azacytidine are able to revive the expression of miR-148a and miR-152 genes and mediate growth inhibition of epithelial ovarian cancer cells. The present study suggests that re-expression of miR-148a and miR-152 by epigenetic therapy aiming to DNMT1 suppression might resensitize resistant ovarian tumors to conventional chemotherapy.

## Introduction

Epithelial ovarian cancer (EOC) is the leading cause of death from gynecological cancers, causing more than 140,000 deaths worldwide annually (1, 2). More than 70% of patients with ovarian cancer are diagnosed at advanced stages due to the lack of specific symptoms when the tumor is limited to the ovaries (3). The standard treatment for patients is surgery to remove most possible extent of cancerous cells followed by platinum-based chemotherapy such as cisplatin chemotherapy alone or in combination with Taxanes (4). Despite high response rate to initial platinum-based chemotherapy, most of the patients relapse within 18 months after first-line chemotherapy. Therefore chemo-resistance is the main limitation of successful treatment for ovarian cancer (5). Finding the agents that are able to reverse chemo-sensitivity to cisplatin can promote long-term survival rate of ovarian cancer patients (6). The term epigenetics refers to heritable alterations in cellular phenotype, independent of changes in the deoxyribonucleic acid (DNA) sequence. Epigenetic mechanisms include histone modifications, DNA methylation, chromatin remodeling, and post-translational regulation of gene expression by micro-ribonucleic acid (miRNA) molecules. DNA methylation and histone deacetylation are two fundamental mechanisms involved in gene regulation and are catalyzed by DNA methyltransferases (DNMTs) and histone deacetylases (HDACs); respectively (7). Epigenetic aberrations are involved in ovarian cancer progression and drug resistance (8). Remarkable increased expression of DNA methyltransferases has been observed in cisplatin-resistant ovarian cancer (9). Despite genetic alterations, epigenetic abnormalities are potentially reversible and can be corrected (8). miRNAs are small endogenous non-coding RNAs that modulate gene expression mainly by binding to 3’UTR of target messenger RNA (mRNA) in order to either mRNA degradation or inhibition of protein translation. A number of miRNAs are related to the sensitivity of ovarian cancer cells to chemotherapeutic agents (10). MiR-152 and miR-148a belong to the same miRNA family and are down-regulated in ovarian cancer (11). The principal mechanism of down-regulation of these miRNAs is hyper-methylation of the promoter region (12). Hyper-methylation in miR-148a-coding region occurs in pancreas, breast and gastric cancers. In addition, high degree of methylation of the miR-152 CpG island is found in mixed-lineage leukemia (MLL), rearranged acute lymphoblastic leukemia, endometrial, and gastrointestinal cancers (13). It is also observed that miR-152 is significantly down-regulated in A2780 and SKOV3 resistant ovarian cancer cell lines. DNMT1, the main enzyme to maintain DNA methylation pattern during DNA replication and repair, is one of the most important validated targets of miR-152 and miR-148a (14). Inducing miR-152 and miR-148a expression can result in down-regulation of DNMT1 and consequently up-regulation of tumor suppressor genes. While DNMT and HDAC inhibitors can separately relieve epigenetic alterations, their synergic effects on silenced gene re-expression have also been verified (15). As a result, combining these two classes of epigenetic drugs with conventional therapies may be an efficient approach in resensitizing to chemotherapeutic agents. In this study for the first time the effect of Trichostatin A and 5-Azacytidine (5-Aza) on miR-148a and miR-152 expression in A2780CP ovarian cancer cell line was investigated. 

## Experimental

*Cell lines and cell culture*

Human epithelial ovarian cancer cell line A2780S (NCBI Code: C461) and its derivative cisplatin resistant A2780CP (NCBI Code: C454) were obtained from Pasteur Institute of Iran. The cell lines were maintained in RPMI1640 medium (Gibco, USA), supplemented with 1% Antibiotic-Antimycotic solution (Inoclon, Iran), and 10% fetal bovine serum (FBS, Inoclon, Iran). Both cell lines were cultured in humidified atmosphere of 5% CO_2_ at 37 ^o^C.

*Chemicals*

5-azacytidine (100mg, powder), Trichostatin A (5µM, ready-made solution), and MTT (3-(4, 5-Dimethyl-2-thiazolyl)-2,5-diphenyl-2H-tetrazolium bromide) dye (100 mg, powder) were provided from Sigma-Aldrich Co.,USA. MTT dye and 5-Aza were dissolved in phosphate buffered saline (PBS, Inoclon, Iran) and dimethyl sulfoxide (DMSO, Inoclon, Iran), respectively and stored at −20 °C.

*Cell viability assay*

Cell viability was evaluated by The 3-(4,5-dimethylthazol-2-yl)-2,5-diphenyltetrazolium bromide (MTT) assay. Briefly, A2780S and A2780CP cells were seeded at a density of 1×10^4 ^cells/well into a 96 well plate and 24 h later incubated with 6 increasing concentrations of TSA (0.05, 0.1, 0.25, 0.5, 1, 2 µM) and 5-Aza (0.5, 1, 2.5, 5, 7.5, 10 µM) for 48 h to determine a dose-response curve. 100 μL MTT (0.5 mg/mL) was supplemented to the appropriate wells, followed by 4 h incubation. The super­natant was then disposed. 100 μL of DMSO was added to each well to dissolve the precipitate. The absorbance of each well was measured using the microplate reader (BioTek Instruments, Inc, USA) at a wave length of 570 nm and 630 nm. Five wells were considered for each concentration, and two independent experiments were performed. Cell Viability was measured at different time points (24, 48 and 72 h), to determine optimal incubation time. Subsequently, the cell lines were treated in 6 well plates with the IC50 dose of each drug, alone and in combination at the optimal time to perform RNA extraction.

*RNA extraction and polyadenylation*

Total RNA was purified using Trizol reagent (Invitrogen, USA) from A2780CP cells. 1 mL of Trizol was added to each well. After 5 min homogenizing and transferring to a microtube, 200 µL of chloroform was added and shaken vigorously; then incubated for three minutes at room temperature. Samples were centrifuged and the aqueous phase (~400 µL) was transferred to a microtube. 500 µL of isopropanol was added and incubated for 10 min. Following centrifugation, pellets were washed with 1 mL of cold 75% ethanol, centrifuged and resuspended in 40 µL of RNase free water. RNA was quantified and complementary DNA (cDNA) was immediately synthesized from total extracted RNA using a RevertAid^TM^ First Strand cDNA Synthesis Kit (Thermo Fisher Scientific, USA). RNA polyadenylation was performed before cDNA synthesis for miRNA analysis. 2µL of total RNA was polyadenylated with ATP by poly (A) polymerase enzyme at 37 °C for 10 min, following the manufacturer’s protocol using a Poly (A) Tailing Kit (ParsGenome, Iran). The poly (A) tailed RNA was used for RT-PCR reaction incubated for 60 min at 44 ^o^C followed by 85 ^o^C for 1 min for enzyme inactivation (according to ParsGenome MiR-Amp kit instructions). cDNA was subse­quently held at -20 °C.

*Real-time quantitative PCR (q-PCR)*

Real-time quantitative PCR was accomplished using Maxima SYBR Green quantitative polymerase chain reaction (q-PCR) Master Mix (Thermo Fisher Scientific, USA). Amplification and detection of miR-148a, miR-152, and DNMT1 were carried out using the ABI 7500 Real-Time PCR system (Applied Biosystems, USA). *GAPDH* and *RNU6B* were considered as endogenous controls for mRNA and miRNA analysis, respectively. Primer sequences were as follows: DNMT1 forward: 5′-CACCAGGCAAACCACCATCAC-3′, DNMT1 reverse: 5′-AGCGGTCTAGCAACTCGTTCTC-3′, GAPDH forward: 5′-TGCACCACCAACTGCTTAGC-3′, GAPDH reverse: 5′-GGCATGGACTGTGGTCATGAG-3′, RNU6B forward: 5′-GCTTCGGCAGCACATATACTAAAAT-3′, RNU6B reverse: 5′-CGCTTCACGAATTTGCGTGTCAT-3′, MiR-148a forward: 5′-ATGCTCAGTGCACTACAGAA-3′, MiR-148a reverse: 5′-GTGCAGGGTCCGAGGT-3′(16), miR-152 forward: 5′-GGCAGTGCATGACAGAAC-3′, miR-152 reverse: 5′-CAGTGCGTGTCGTGGAGT-3′(14). The final volume of RT-qPCR reaction was 25 µL containing 12.5 µL SYBR Green Master Mix Reagent, Forward and reverse primers, cDNA (1µL) and DNase free water up to 25µL. The PCR amplification condition of DNMT1 consists of 40 cycles at 95 °C for 5 seconds, 60 °C for 30 seconds, and 72 °C for 30 seconds. The PCR amplification condition of miR-148a and miR-152 consists of 40 cycles at 95 °C for 5 seconds, 64 °C for 20 seconds, and 72 °C for 30 seconds. The fold changes for expression of each gene were evaluated by the comparative threshold cycle (Ct) method using the formula 2^-(∆∆Ct)^.

*Statistical analysis*

Statistical Package for the Social Sciences (SPSS version 22.0, USA) Software and GraphPad Prism 7.0 (GraphPad Software, USA) were used for analyzing the data and designing the graphs respectively. The differences between experimental groups were assessed using one-way analysis of variance (ANOVA), and the significance level was considered *P *≤ 0.05.

## Results

*A2780CP is resistant to Cisplatin-induced cell death*

**Figure 1:**
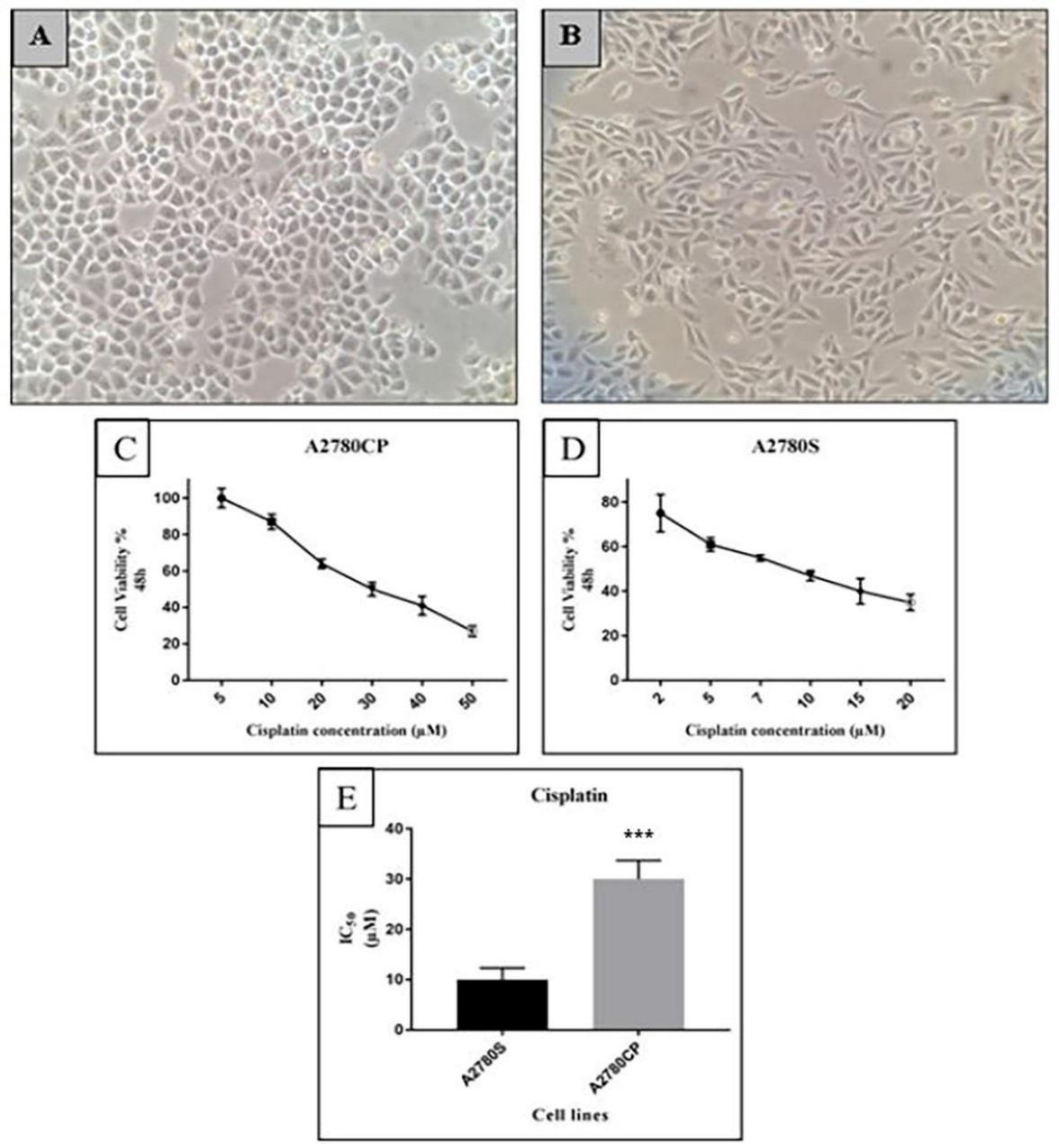
Morphology of A) A2780CP Cell Line and; B) A2780S Cell Line, 10x magnification. Determination of Cisplatin-mediated cell viability in ovarian cancer cell lines; C) A2780CP and; D) A2780S cells were subjected to a concentration gradient of cisplatin for 48 h and IC50 was determined by the MTT assay. The cell viability in terms of untreated control cells was expressed as percentages; E) The IC50 of cisplatin in both cell lines was calculated and compared. The IC50 of cisplatin for A2780CP cells was 3 fold more than the IC50 for A2780S cells. Each value represents the means ± standard deviation (SD), ***P ≤ 0.001

We verified the cisplatin resistance of A2780CP cell line compared with its cisplatin-sensitive parental A2780 cells by performing an MTT assay. As illustrated in the figure1, A2780CP cells have a round shape, while A2780S cells have a spindly appearance and their proliferation activity is very high in comparison with A2780CP cells (Figures 1A and 1B). A2780S and A2780CP Cells were exposed to increasing concentrations of cisplatin (2-20 µM and 5-50 µM respectively) for 48 h. Dose response curve was plotted subsequently (Figures 1C and 1D). As expected, A2780CP cells were more resistant than A2780S cells (3-fold) (Figure 1E).

*Determination of half maximal inhibitory concentration of 5-Aza and TSA*

**Figure 2:**
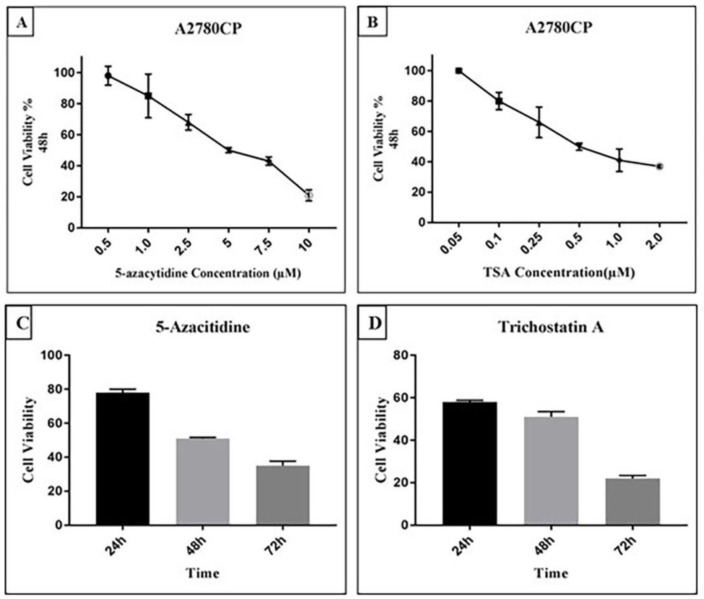
5-Aza and TSA both inhibit the growth of A2780CP cells in a concentration and time-dependent manner.Cell viability of A2780CP cells was measured using MTT assay in the presence of TSA and 5- Aza. Cells were treated with various concentrations of **A**) 5-Aza and; **B**) TSA for 48 h. Cell viability in terms of untreated control cells was expressed as percentages. The IC50 obtained for 5-Aza and TSA was 5 µM and 0.5 µM respectively. Time course of drug treatment at a single dose of 5-Aza and TSA in A2780CP cell line was determined by MTT assay when cells were treated with **C**) 5-Aza (5 µM) and; D) TSA (0.5 µM), in different time points (from 24 to 72 h). treated cells displayed maximum growth inhibition in 72 h treatment

Cell viability of A2780CP cells in the presence of TSA and 5-Aza was assessed by MTT Assay. Dose response curves for two drugs were designed with doses ranging from 0.5 µM to 10 µM for 5-Aza and 0.05 µM to 2 µM for TSA at 48 h (Figure 2A and 2B). The concentration leading to a 50% decrease in cell viability (IC_50_) was found to be 0.5 and 5 µM for TSA and 5-Aza respectively. To evaluate whether cell viability of A2780CP cells is affected by the exposure time of the drugs, a time course assay (24, 48 and 72 h) was performed. Forty-eight hours was determined to be the optimal time of TSA and 5-Aza exposure (Figure 2C and 2D). The results of MTT assay demonstrated that 5-Aza and TSA mediate growth arrest of A2780CP cells in a concentration- and time-dependent manner.

*Re-sensitivity of A2780CP cells to Cisplatin after treatment with TSA and 5-Aza*

**Figure 3:**
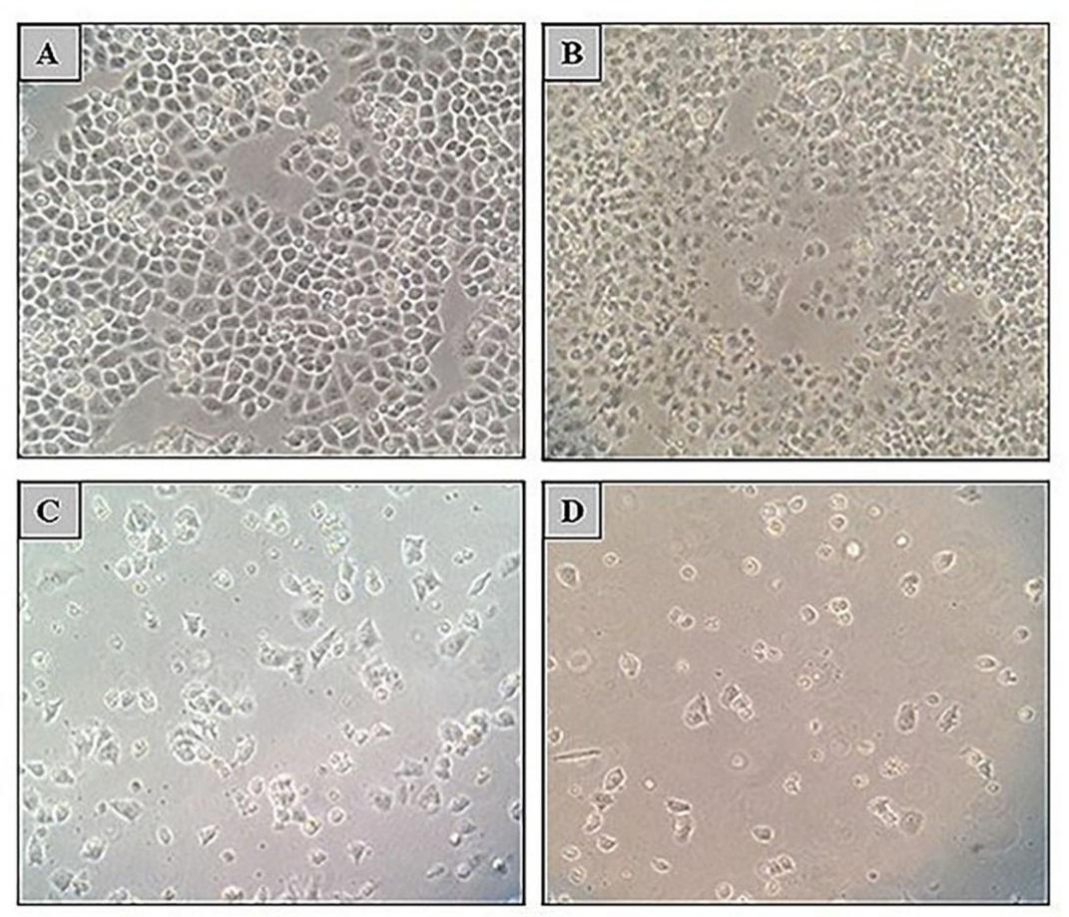
Effect of TSA, 5-Aza, and cisplatin alone or in combination on cell survival and morphology of A2780CP cell line. Morphology of A2780CP cells was observed using an optical inverted microscope (magnification, 10X). A) Treatment with 10 µM cisplatin could not inhibit A2780CP cells growth (96% cell viability) whereas; B) 48 h treatment with 5-Aza (5 µM); C) 72 h treatment with 5-Aza (5 µM) and; D) 48 h co-treatment with 5-Aza (5 µM) and TSA (0.5 µM) following 48 h with cisplatin could result in growth inhibition (65%, 33% and 12% cell viability; recpectively).

A2780CP cell line was treated with 5 µM of 5-Aza and 0.5 µM of TSA separately and in combination followed by 10 µM of cisplatin. As expected, A2780CP cells were resistant to Cisplatin toxicity, but the cells were resensitized to cisplatin-mediated cytotoxicity after treatment with TSA or/and 5-Aza (Figure 3).

*The effect of TSA or 5-Aza on miR-148a and miR-152 expression*

**Figure 4:**
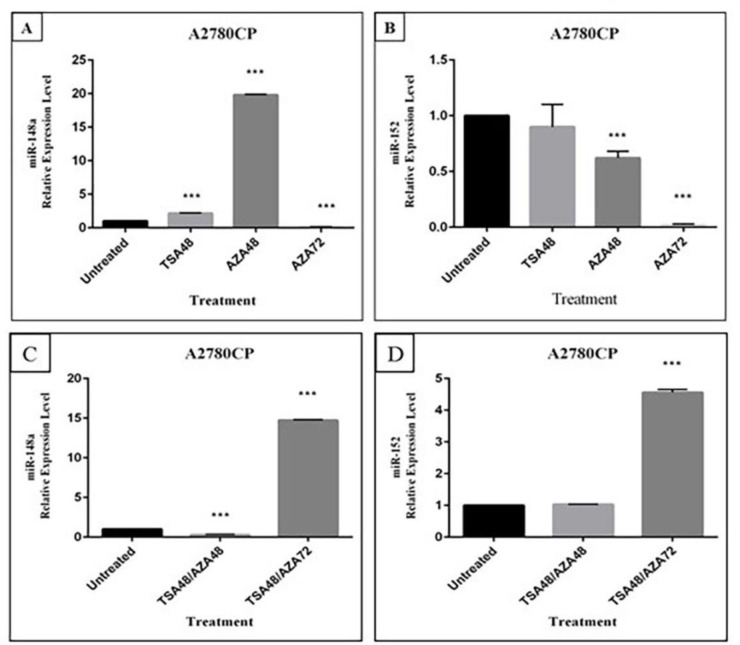
Effect of TSA, and 5-Aza on miR-148a and miR-152 expression. A2780CP cells were treated with 0.5 µM TSA for 48 h and 5 µM 5-Aza for 48 h and 72 h separately and expression of A) miR-148a and; B) miR-152 was assessed by Real time PCR.Combination effect of TSA and 5-Aza on expression of C) miR-148a and; D) miR-152 was measured after cells were treated with a combination of 0.5 µM TSA for 48 h and 5 µM 5-Aza for 48 h and 72 h. RNU6B was considered as the internal control. Values represent mean±SD, for four independent experiments, ***P ≤ 0.001

To evaluate the effect of TSA or 5-Aza on miR-152 and miR-148a expression, A2780CP cells were treated with these drugs for 48 h. RT-qPCR analyses indicated that miR-148a expression in A2780CP cells was about 2-fold up-regulated by TSA compared with control DMSO-treated cells as shown in Figure 4A, while treatment with TSA could not revive miR-152 expression (Figure 4B). The expression of miR-148a was significantly up-regulated after 48 h treatment with 5-Aza (19.8 fold). On the other hand, there was no increase in miR-152 expression in comparison with control DMSO-treated cells. An additional 72 h treatment with 5-Aza was carried out. However, no up-regulation of miR-148a and miR-152 expression was observed.

*The effect of TSA and 5-Aza combination on miR-148a and miR-152 expression*

The effect of a simultaneous exposure of A2780CP cells to 5-Aza and TSA was investigated to evaluate whether the combination of both agents induces a greater reactivation of miR-152 and miR-148a than treatment with each one alone. Up-regulation of both miR-152 and miR-148a was only observed after co-treatment with TSA for 48 h and 5-Aza for 72 h (Figures 4C and 4D).

*DNMT1 expression in A2780-CP cell line after treatment with TSA and 5-Aza*

**Figure 5:**
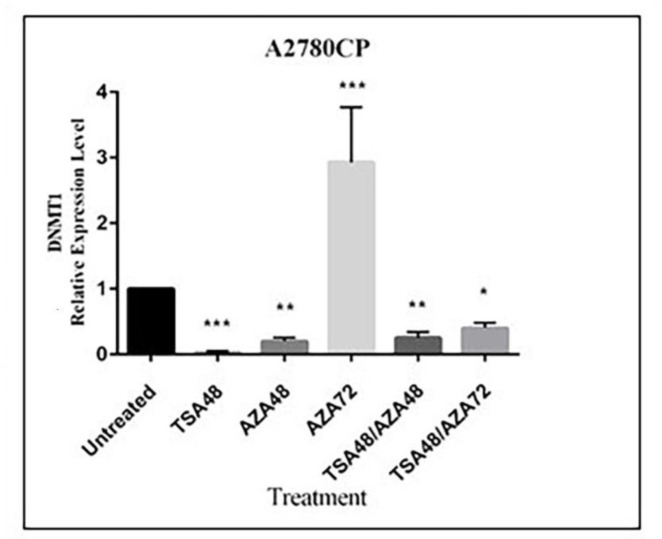
DNMT1 expression in A2780CP cell line after treatment with TSA and 5-Aza. A2780CP cells were treated separately or in combination with 0.5 µM TSA for 48 h and 5 µM 5-Aza for 4 h and 72 h. Expression of DNMT1 and GAPDH (as the internal control) was assessed by Real time PCR. Values represent mean ± standard deviation (SD), for four independent experiments *P ≤ 0.05, **P ≤ 0.01, ***P ≤ 0.001

DNMT1 expression was significantly suppressed in A2780CP cells when treated with TSA or 5-Aza for 48 h alone, while a 3-fold up-regulation was observed in 5-Aza treatment for 72 h. Furthermore, DNMT1 expression was decreased in co-treatment with TSA for 48 h and 5-Aza for 48 h or 72 h (Figure 5).

## Discussion

Cisplatin is the first-line chemotherapeutic agent for many malignancies including ovarian cancer. Cisplatin forms DNA adducts which inhibit DNA and RNA polymerases, interfere with DNA replication and transcription, and ultimately trigger apoptosis (17). Ovarian cancer is the most common gynecological malignancy after breast cancer in women older than 40 years old particularly in developed countries (18). One of the most promising strategies for overcoming chemoresistance in ovarian cancer is combining cisplatin therapy with other agents that enhance the anticancer effect of cisplatin (14).

DNA hypermethylation and histone deacetylation have a major role in ovarian cancer chemo resistance, through silencing of drug resistance associated genes. Therefore, the inhibition of these epigenetic pathways can result in gene re-expression and consequently resensitization to chemotherapeutic agents (8). Our results revealed that 5-Aza and TSA can inhibit growth of ovarian cancer A2780 cells in a concentration and time dependent manner. 5-Azacytidine is a DNA methyltransferase inhibitor that was approved in 2004 by the Food and Drug Administration (FDA) for the treatment of myelodysplastic syndromes (MDS). 5-Aza can decrease the DNA methylation level and regulate the gene expression through reducing the bioactivity of DNA methyltransferase enzymes by trapping them for proteasomal degradation (19). TSA is an antifungal antibiotic which inhibits class I and II HDAC enzymes by chelating zinc ion at the active site (20). Previous studies indicated that histone deacetylation in hyper-methylated genes could result in gene inactivation while acetylation of histones could not lead to re-expression of silenced genes (21). It has been claimed that TSA promotes Bax-dependent apoptosis in cisplatin-resistant ovarian cancer cells by up-regulation of p-73 (22).

 MiRNAs are small regulatory RNAs that are aberrantly expressed in different cancer types; (23) and their role is demonstrated in the development of drug resistance (24). A number of studies have asserted that overexpression of some specific miRNAs can enhance the sensitivity of ovarian cancer cells to chemotherapeutic agents. Chen *et al* reported that transfection of miR-133b into paclitaxel and cisplatin resistant ovarian cancer cell lines results in increased sensitivity to paclitaxel and cisplatin (25). In the other study, treatment with miR-142-5p mimic enhanced cisplatin-induced apoptosis in ovarian cancer cells (26).

Many evidences show that miR148/152 family members act as tumor suppressor miRNAs and are down-regulated in many types of cancers as well as ovarian cancer (13). MiR-148a targets various genes which affect a series of biological processes such as proliferation (IGF-IR, ROCK1, IRS1 and ERBB3), apoptosis (BCL-2), metastasis, and invasion (SMAD2, WNT1/10B, and USP4) (27). Additionally, MET, TGFα, FGF2, CD151, and MMP3 are some of the oncogenic targets of miR-152 in human cancers; by targeting these genes, miR-152 leads to inhibition of cell proliferation and tumor metastasis (12). It has been demonstrated that decreased expression of miR148a/152 is in association with increased tumor size and stages in gastric cancer (28). It has also been observed that the lower expression of these miRNAs is inversely correlated with stage and lymph node status of breast cancer tumors (29). Down-regulation of miR-148a and miR-152 leads to overexpression of DNMT1, promoting DNA methylation which could result in suppression of genes involved in chemo-resistance. Several genes silenced by promoter DNA hypermethylation such as MLH1, SULF1, SFRP, TNFRSF10A, UCHL1, and CLDN4 are related to platinum resistance in ovarian cancer. These genes are principally involved in DNA repair, apoptosis, cell growth, and invasion (30). Restoration of these tumor suppressor genes could contribute to the re-sensitization of resistant ovarian cancer cells to platinum-based chemotherapeutic agents. Down-regulation of DNMT1 as a direct target of miR-148a and miR-152 by a negative feedback can result in higher expression of these miRNAs. Such a regulatory circuit has been reported in breast cancer; (29) and might exist in ovarian cancer as well. In the present study, expression levels of miR-148a and miR-152 in A2780CP ovarian cancer cell line was investigated after treatment with TSA and 5-Aza using Real-Time PCR. We hypothesized that up-regulation of miR-148a and miR-152 by treatment with 5-Aza and TSA could reverse cisplatin resistance through DNMT1 suppression in ovarian cancer and miR-148a and miR-152 down-regulation might be due to DNA methylation and histone de-acetylation. DNMTs and HDACs strengthen the effect of each other in transcriptional suppression. Methylated DNA recruits HDACs either through methyl-CpG binding proteins or directly by DNMTs (31). Therefore, combination use of DNMTi and HDACi may improve the limited anticancer efficacy observed with either therapeutic class alone. We observed that miR-148a was re-expressed in cisplatin-resistant cells when treated with 5-Aza and TSA alone and in combination; while miR-152 was only re-expressed when treated with a combination of these two drugs in comparison with control group. Surprisingly, miR-148a up-regulation after treatment with TSA for 48 h and 5-Aza for 72 h in association, was lower than treatment with 5-Aza for 48 h alone. These data indicate the potential of 5-Aza and TSA to reverse the expression of miR-148a and miR-152. The study by Cacan* et al.*, consistent with our observations, demonstrated that 5-aza-CdR and TSA treatment both enhance RGS10 (an important regulator of chemoresistance in ovarian cancer) expression and cisplatin-mediated cell death significantly in A2780CP ovarian cancer cell line alone and in combination. Further investigation demonstrated the contribution of HDAC1 and DNMT1 to silencing of RGS10 during acquired chemo-resistance and revealed the importance of HDAC1 and DNMT1 inhibition as an assistant therapeutic approach to overcome ovarian cancer chemo-resistance (32). In another study on endometrial cancer cell lines it was revealed that hypermethylated miR-137 was overexpressed after co-treatment with 5-aza-CdR and TSA, more effective than treatment with each epidrug individually (33). Liu *et al* ,found that TSA and TSA plus 5-Aza treatment could significantly up-regulate RhoB and mediate apoptosis of SKOV3 and A2780 ovarian cancer cells, while 5-Aza could not do it alone (34). In a recent study it was reported that 5-aza-CdR and TSA can significantly downregulate DNMT1 and HDAC1 and upregulate p21, p27, and p57 genes expression in colon cancer SW480 cell line separately but their joint application was not investigated (35).

We demonstrated that miR-152 and miR-148a in cisplatin resistant A2780 cell lines were up-regulated after treatment with 5-Aza and TSA and consequently down-regulation of DNMT1 was observed. Even though DNMT1 is a direct target of miR-148a and miR-152, treatment-mediated up-regulation of the other hyper-methylated miRNAs targeting DNMT1 such as miR-185 on down-regulation of this gene should not be ignored. Xiang *et al* ,revealed that over-expression of miR-152 and miR-185 leads to cisplatin sensitivity mainly through direct targeting of DNMT1(14). We observed no significant up-regulation in miR-152 expression after treatment with DNMTi, 5-Aza. Our result is in consistent with their study that reported no significant change in miR-152 expression after treatment with 5-aza-CdR, a nucleoside analog inhibitor of DNA methyltransferases, in cisplatin-resistant A2780 and SKOV3 ovarian cancer cell lines. DNMT1 expression was interestingly down-regulated after 72 h treatment with 5-Aza; such an effect could be probably resulted from de-methylation of hyper-methylated transcription factors, after treatment with 5-Aza which consequently induces the expression of DNMT1. Examples of such hyper-methylated transcription factor genes are HOXA5; (36) and members of the GATA family (37). Findings of this study suggest an understanding of mechanisms by which epigenetic therapeutic options might provide a new approach to the treatment of ovarian chemo-resistance. In a recent study it was determined that combining DNMTi and HDAC6i upregulates expression of interferon stimulated genes and cytokines significantly more than each epigenetic drug individually in several cisplatin-resistant ovarian cancer cell lines. Thus it increases anti-tumor immune signaling from cancer cells (38). Combined with platinum-based agents, DNA methyltransferase, and histone deacetylases inhibitors can sensitize resistant ovarian cancer cell lines to these chemotherapeutic agents and the same effect may be observed in animal models and the patients with recurrent ovarian cancer. Although the present study focused on miR-152 and miR-148a, some other microRNAs are likely to be involved in cisplatin resistance.

## Conclusion

In conclusion, in this study we demonstrated that the two epigenetic modifiers, TSA and 5-Aza, depending on treatment condition (time of exposure and applying epidrugs alone or in combination), can result in re-expression of miR-148a and miR-152 in cisplatin-resistant A2780CP ovarian cancer cells.

Therapeutic strategies targeting specific miRNAs based on epigenetic intervention might provide novel approaches for cancer therapy in the future. The nonspecific activation of genes by epidrugs may limit clinical application of these agents; however designating an optimal drug administration is necessary to achieve an optimal treatment outcome.
